# Microbial nitrogen transformations in tundra soil depend on interactive effects of seasonality and plant functional types

**DOI:** 10.1007/s10533-024-01176-6

**Published:** 2024-08-30

**Authors:** Marianne Koranda, Anders Michelsen

**Affiliations:** 1 Division of Terrestrial Ecosystem Research, Centre for Microbiology and Environmental Systems Science, University of Vienna, Djerassiplatz 1, 1030 Vienna, Austria; 2 Terrestrial Ecology Section, Department of Biology, University of Copenhagen, 2100 Copenhagen, Denmark; 3 Center for Permafrost (CENPERM), University of Copenhagen, 1350 Copenhagen, Denmark

**Keywords:** N mineralization, Nitrification, Protein depolymerisation, Plant functional types, Soil N-cycling, Tundra

## Abstract

Nitrogen (N) cycling in organic tundra soil is characterised by pronounced seasonal dynamics and strong influence of the dominant plant functional types. Such patterns in soil N-cycling have mostly been investigated by the analysis of soil N-pools and net N mineralisation rates, which, however, yield little information on soil N-fluxes. In this study we investigated microbial gross N-transformations, as well as concentrations of plant available N-forms in soils under two dominant plant functional types in tundra heath, dwarf shrubs and mosses, in subarctic Northern Sweden. We collected organic soil under three dwarf shrub species of distinct growth form and three moss species in early and late growing season. Our results showed that moss sites were characterised by significantly higher microbial N-cycling rates and soil N-availability than shrub sites. Protein depolymerisation, the greatest soil N-flux, as well as gross nitrification rates generally did not vary significantly between early and late growing season, whereas gross N mineralisation rates and inorganic N availability markedly dropped in late summer at most sites. The magnitude of the seasonal changes in N-cycling, however, clearly differed among plant functional types, indicating interactive effects of seasonality and plant species on soil N-cycling. Our study highlights that the spatial variation and seasonal dynamics of microbial N transformations and soil N availability in tundra heath are intimately linked with the distinct influence of plant functional types on soil microbial activity and the plant species-specific patterns of nutrient uptake and carbon assimilation. This suggests potential strong impacts of future global change-induced shifts in plant community composition on soil N-cycling in tundra ecosystems.

**Supplementary Information:**

The online version contains supplementary material available at 10.1007/s10533-024-01176-6.

## Introduction

Nitrogen (N) cycling in organic tundra soils is characterised by strong N limitation (Sistla et al. 2012; Wild et al. 2015), as well as pronounced seasonal dynamics (Grogan and Jonasson 2003; Schimel et al. 2004; Edwards and Jefferies 2013). The seasonality of the soil N-cycle is driven by environmental conditions, the seasonality of plant activity and their influence on microbial substrate availability: The winter period, typically a phase of increasing microbial biomass and positive net N mineralisation rates, is succeeded by a sudden crash in microbial biomass and subsequent pulse release of microbial metabolites during snow melt in spring (Lipson et al. 1999; Schmidt et al. 2004; Edwards et al. 2006). This decline in microbial biomass is presumably due to microbial substrate depletion (Lipson et al. 2000; Buckeridge and Grogan 2008), microbial lysis as a result of freeze–thaw events or changes in osmotic potential (Larsen et al. 2002; Henry 2007; Sorensen et al. 2018), as well as microbial community changes (Wallenstein et al. 2007). The resulting spring pulse in dissolved N fuels plant N uptake at the onset of plant growth in early growing season (Larsen et al. 2012). The following summer period is characterised by strong competition for N between plants and soil microbes, causing very low levels of soil N availability and microbial net N immobilization, before microbial substrate availability again increases upon litterfall in autumn (Weintraub and Schimel 2005a; Edwards and Jefferies 2013; McLaren et al. 2017).

Most studies on soil N cycling include measurements of dissolved N availability, microbial biomass and net N mineralization rates (e.g., Schmidt et al. 2002; Bardgett et al. 2007; Edwards and Jefferies 2013). These parameters, however, only insufficiently describe soil N cycling, as concentrations of low molecular weight N compounds in soil solution result from the equilibrium of several processes, mainly proteolytic enzyme activities (i.e., decomposition of soil organic matter and turnover of microbial biomass N), microbial N uptake and release, plant N uptake, interactions with the soil matrix and ecosystem N losses (Hart et al. 1994; Schimel and Bennett 2004; Jilling et al. 2018). A more thorough analysis of soil N dynamics hence requires measurements of microbial gross N cycling processes, e.g. by the ^15^N—pool dilution technique (Davidson et al. 1992; Braun et al. 2018), which is, however, relatively seldom applied, likely because of the time effort of the method. Only very few studies have analysed gross N cycling processes in arctic or alpine tundra ecosystems at more than one time point (e.g., Fisk et al. 1998; Weintraub and Schimel 2005b; Xu et al. 2021), which allows the analysis of seasonal patterns in N dynamics.

Besides the effect of seasonality, soil N availability and microbial N transformations are strongly dependent on the dominant plant species. Plants influence soil N cycling not only directly via root N uptake and plant-specific differences in timing of N uptake or preference for N-forms (McKane et al. 2002; Larsen et al. 2012; Pedersen et al. 2020), they also affect soil microbial processes via their impact on the composition and activity of soil microbial communities (Eskelinen et al. 2009; Gavazov et al. 2022; Koranda et al. 2023), via plant litter quality and impact on the soil organic matter quality (Dorrepaal et al. 2005; Adamczyk et al. 2016; Koranda et al. 2023). Very few studies have considered effects of plant community composition on gross N cycling in high-latitude ecosystems by comparing different vegetation types (Buckeridge et al. 2010; Marushchak et al. 2011; Ramm et al. 2022b) or by analysing microsites characterised by different plant communities (Biasi et al. 2005). Studies explicitly investigating effects of plant species or plant functional types on soil microbial N transformations are, however, to our knowledge still lacking.

In this study we addressed this knowledge gap by comparing the effects of two dominant plant functional types in tundra heath, dwarf shrubs and mosses, on microbial N transformations and soil N availability. Dwarf shrubs and bryophytes are abundant in many arctic plant communities, but have been shown to react differently to climate change: While an expansion of (deciduous) shrubs has been observed in recent decades as a result of the warming climate, the abundance of bryophytes tends to decline (Elmendorf et al. 2012; Alatalo et al. 2020; Mekonnen et al. 2021). Bryophytes have often been ignored in studies on ecosystem carbon and nutrient cycling, despite their important role in the functioning of high-latitude ecosystems (Koranda and Michelsen 2021; Slate et al. 2024; Turetsky 2003). For this study, we selected three moss species differing in morphology and habitat preference (*Hylocomium splendens, Aulacomnium turgidum and Tomentypnum nitens*). All three moss species are known to host N-fixing bacteria (Rousk et al. 2013; Stuart et al. 2021). Furthermore, we selected three dwarf shrub species of distinct growth form and mycorrhizal association: *Empetrum hermaphroditum* (evergreen, ericoid mycorrhiza), *Arctostaphylos alpinus* (deciduous, arbutoid mycorrhiza) and *Betula nana* (deciduous, ectomycorrhiza). We collected soil samples at the beginning and end of the growing season, as the influence of plant functional traits on soil N cycling can be expected to be most pronounced during the period of high plant activity in summer. We aimed at elucidating (1) how microbial N-cycling processes and concentrations of plant-available N forms vary between early and late growing season; (2) how these patterns are influenced by plant species and plant functional types. We tested three main hypotheses: (i) We hypothesized that at moss-grown sites, dissolved N availability and gross N-cycling rates would be high in early growing season, due to the lasting effect of the spring peak in soil N availability, and that N availability would decrease in late growing season. (ii) At shrub sites, on the contrary, we expected that soil N availability and N-cycling rates would be low in early growing season, during the period of most intensive plant N-uptake, and that N availability would increase until end of summer. (iii) We further hypothesized that the seasonal variation in soil N availability and N cycling rates would be more pronounced under deciduous shrubs than under evergreen shrubs, as the latter exhibit more constant N uptake rates and photosynthetic activity over the growing season.

## Materials and methods

### Study site

The study was performed in a tundra heath located close to Abisko in subarctic Sweden (68° 20′ 24.7ʺ N, 18° 50′ 35.5ʺ E). We chose a study site characterised as erect dwarf-shrub, moss tundra (Circumpolar Arctic Vegetation Map, Raynolds et al. 2019), located below the tree line and surrounded by open mountain birch forest (*Betula pubescens*). Vegetation at the study site was dominated by evergreen and deciduous dwarf shrubs (*Empetrum hermaphroditum*, *Arctostaphylos alpinus*, *Vaccinium uliginosum*, *Betula nana, Salix sp.*) and mosses (*Hylocomium splendens*, *Aulacomnium turgidum*, *Tomentypnum nitens, Sphagnum fuscum*), scattered grasses, sedges and forbs. The vegetation structure was characterised by a patchy distribution of plant species. In contrast to other tundra types (e.g., tussock tundra), the distribution of plant species at the study site was not strictly related to microtopography, although some microsite preferences of plant species were apparent (i.e., troughs and wetter sites were preferentially grown by mosses, and hummocks were dominated by ericaceous shrubs). Soil type was classified as histosol, consisting of an organic horizon of 8–12 cm depth underlain by glacial till (see Table 1 for detailed soil characteristics). Mineral horizon was mostly absent or very shallow. Bedrock in the Abisko region consists of mica schists with dolomite outcrops. Soils at the study site were underlain by discontinuous permafrost. Yearly precipitation for 2017–2018 was 340 mm and mean annual temperature was 0.2 °C (climate data from Abisko Research Station).

**Table 1:** Soil characteristics at sites grown by the dwarf shrub species *Empetrum hermaphroditum, Arctostaphylos alpinus and Betula nana* and the moss species *Hylocomium splendens, Aulacomnium turgidum and Tomentypnum nitens*

	C (%)		N (%)		C:N ratio		Soil moisture (% of FW)		pH-value		Bulk density (g DW cm^−3^)		Organic horizon depth (cm)	
*E. hermaphr.*	49.1	(0.3)^a^	1.23	(0.01)^a^	40	(0)^a^	76.0	(1.0)^ab^	5.1	(0.1)^a^	0.105	(0.010)^a^	8.1	(0.3)
*A. alpinus*	48.6	(0.6)^a^	1.31	(0.10)^ab^	38	(3)^a^	75.4	(1.0)^a^	5.3	(0.3)^a^	0.107	(0.007)^a^	9.5	(0.8)
*B. nana*	45.7	(1.8)^ab^	1.29	(0.03)^ab^	36	(2)^ab^	74.6	(1.0)^a^	5.7	(0.2)^a^	0.092	(0.009)^ab^	10.3	(0.7)
*H. splendens*	44.3	(1.8)^ab^	1.46	(0.06)^ab^	31	(1)^b^	76.0	(1.0)^ab^	6.4	(0.2)^b^	0.084	(0.008)^ab^	10.2	(0.5)
*A. turgidum*	43.5	(1.4)^ab^	1.51	(0.08)^b^	29	(2)^b^	79.0	(0.5)^b^	6.9	(0.2)^b^	0.061	(0.007)^b^	9.3	(0.4)
*T. nitens*	40.8	(3.2)^b^	1.38	(0.09)^ab^	30	(3)^b^	77.6	(1.6)^ab^	7.0	(0.0)^b^	0.066	(0.012)^b^	9.8	(0.6)

Values are means (SE in parentheses), n = 5. Groups not sharing the same letter are significantly different (p < 0.05, Tukey’s post-hoc test). Values are means of two soil samplings. Data from Koranda et al. (2023)

### Soil samplings

Five replicate blocks (size between 10 × 10 m and 20 × 20 m) with similar vegetation composition were selected within an area of 4 ha.

Soil samplings were performed on August 29th and 30th 2017 (late growing season) and on July 3rd and 4th 2018 (early growing season). Leaves of the deciduous plant species were already fully developed in late June. The time point end of August corresponded with the start of leaf senescence (or right before the start of senescence, depending on the plant species). At each time point soil cores (4 cm diameter) of the entire organic horizon (8–12 cm deep, entire depth was pooled) were taken under three dwarf shrub species (*Empetrum hermaphroditum*, *Arctostaphylos alpinus*, *Betula nana*) and three moss species (*Hylocomium splendens*, *Aulacomnium turgidum*, *Tomentypnum nitens*). The cover of the respective target plant species at the sampling sites was generally > 90%. In each of the five replicate blocks three subsamples (soil cores) per plant species were taken and bulked. Soil cores of the second sampling campaign were taken close to those of the first sampling (ca. 10 cm distance), in order to avoid spatial variability blurring seasonal differences. Regarding soil cores from moss grown sites, we defined soil as beginning from the zone of partly decomposed moss, which was usually separated from the top layer of undecomposed brown moss by a clearly identifiable border. Soil cores from moss sites typically exhibited a gradient of increasing degree of decomposition and increasing darkness in brown to black colour downwards in the organic horizon. After soil sampling coarse roots (> 1 mm diameter) and visible fine roots (< 1 mm diameter) were removed, soil was homogenized by hand and stored at 4 °C until further analyses. Soil extractions were performed within two days and N cycling assays were performed within three days after soil samplings.

### Concentrations of plant-available N forms

Subsamples of fresh soil were extracted with 0.5 M K_2_SO_4_ (1:10, w/w) and filtered through ash free paper filters (Whatman nr. 42). Concentrations of NH_4_^+^ and NO_3_^−^ in soil extracts were determined by flow-injection analysis (Fiastar 5000, FOSS analytical, Höganäs, Sweden), using applications AN 5220 for NH_4_^+^ and AN5201 for NO_3_^−^, respectively. Concentrations of total free amino acids were analysed fluorometrically using a modified method after Jones et al. (2002) and Darrouzet-Nardi et al. (2013). Briefly, the OPAME-reagent was prepared from o-phthaldialdehyde (OPA), methanol and 3-mercaptopropionic acid and mixed with 0.02 M potassium tetraborate buffer (pH 9.5). 50 µL of samples and 200 µL of reagent solution were then pipetted into black microtiter plates, and fluorescence intensity was measured after 1.5 h at 360 nm excitation wavelength and 460 nm emission wavelength using a Synergy HTX microplate reader (Bio-Tek Inc.). Background fluorescence of samples was measured from samples amended with buffer solution. Standard curves were prepared using leucine in different concentrations. As ammonium also generates fluorescent derivatives with the OPAME-reagent, fluorescence of samples was corrected for ammonium fluorescence by including NH_4_^+^ standard curves and subtracting the fluorescence originating from NH_4_^+^ from total sample fluorescence.

### Protein depolymerisation

Proteolytic enzyme activity was determined by a modified method after Weintraub and Schimel (2005b). The method is based on the addition of toluene to soil slurries, which inhibits microbial uptake of enzymatic reaction products and hence causes accumulation of amino acids in the soil slurry. Soil slurries were prepared from 4 g of fresh soil and 40 mL of water and mixed with 400 µL of toluene. Soil slurries were then incubated at 10 °C, and subsamples of the slurries (1 mL) were taken after 15 min, 4 h and 6 h. Subsamples were immediately mixed with 1 mL of TCA-solution (trichloroacetic acid/acetate buffer) to stop the proteolytic activity and subsequently stored frozen. Upon thawing, samples were centrifuged, and amino acid concentration in the supernatant was determined by the OPAME-method described above.

### Gross N mineralisation and nitrification

Gross N mineralisation and nitrification rates were assessed using the ^15^N-pool dilution method (Kirkham and Bartholomew 1954; Braun et al. 2018). The principle of the method is the addition of ^15^N-labelled NH_4_^+^ or NO_3_^−^, respectively, to the soil N pool, which is then diluted by ongoing microbial N mineralisation and nitrification. This allows the estimation of both production and consumption processes of the ammonium and nitrate pools, respectively.

For the N mineralisation assay, 500 µL of ^15^NH_4_Cl-solution (0.13 mM, 99% ^15^N, ~ 1.8 µg N g^−1^ soil DW) were applied to duplicates of fresh soil (2 g). For the nitrification assay, 500 µL of K^15^NO_3_-solution (0.13 mM, 99% ^15^N) was added in the same way. Soil samples were incubated at 10 °C for 4 h and 24 h, respectively, and extracted with 2 M KCl. For determination of N mineralisation rates, NH_4_^+^ in soil extracts was diffused into acid traps consisting of acidified paper filter discs sealed in Teflon tapes. Acid traps were dried and subsequently analysed by an Eurovector elemental analyzer coupled to an Isoprime IRMS. Due to technical problems with IRMS analyses of one sample batch, several data points of gross N mineralisation had to be excluded, resulting in four instead of five replicates for shrub soils. For determination of nitrification rates, NH_4_^+^ in soil extracts was removed by addition of MgO, then NO_3_^−^ was converted to NH_4_^+^ by adding Devarda’s alloy, and subsequently NH_4_^+^ was analysed by microdiffusion as described above.

Gross N mineralisation / nitrification rates and gross NH_4_^+^/NO_3_^−^ consumption rates were calculated according to the following equations (Kirkham and Bartholomew 1954):

$${\text{grossmin}} = \left( {{\text{A}}_{{\text{t}}} {-}{\text{A}}_{0} } \right)/{\text{t}}*\left( {{\text{ln}}\left( {{\text{APE}}_{0} /{\text{APE}}_{{\text{t}}} } \right)/{\text{ln}}\left( {{\text{A}}_{{\text{t}}} /{\text{A}}_{0} } \right)} \right)$$

$${\text{netmin}} = \left( {{\text{A}}_{{\text{t}}} {-}{\text{A}}_{0} } \right)/{\text{t}}$$

$${\text{grossammcons}} = {\text{grossmin}} - {\text{netmin}}$$

where grossmin is gross N mineralisation, netmin is net N mineralisation, grossammcons is gross NH_4_^+^ consumption. A_t_ is the NH_4_^+^-N pool after time t, A_0_ is the initial NH_4_^+^-N pool, APE (atom percent excess) is atom %^15^N-NH_4_^+^_sample_ – atom %^15^N-NH_4_^+^_background_. For nitrification, NH_4_^+^ is replaced by NO_3_^−^ in the equations.

Gross ammonium consumption rates integrate all NH_4_^+^ consuming processes including microbial NH_4_^+^ immobilisation and nitrification. Gross nitrate consumption rates sum up microbial NO_3_^−^ immobilisation and N losses via denitrification. It should be noted that consumption rates may be stimulated by the ^15^N addition in the assays. However, this effect is generally strongest directly after addition of the ^15^N (Braun et al. 2018), while after 4 h (i.e., timepoint 0 of the assays) processes should be more equilibrated. Furthermore, it is possible that microbial gross N cycling processes were stimulated by homogenizing of soil samples in our assays and thus the determined rates might be overestimated compared to in-situ N-cycling rates. Performing the assays with homogenized soil samples (and not intact soil cores), however, has the advantages that (i) the two soil subsamples are similar in N concentrations and microbial community composition, which is especially relevant in heterogenous soils like tundra soils (ii) homogeneous distribution of the added ^15^N is ensured, which is necessary for accurate calculations of N transformation rates.

### Turnover times of plant-available N pools

Estimated turnover times of the pools of total free amino acids, ammonium and nitrate, respectively, in soil were calculated by dividing the pool size of the respective N pool by the average of production and consumption rate (e.g., gross N mineralisation and NH_4_^+^ consumption rate). In case of amino acid turnover, we used only the amino acid production rates for the calculations, as consumption rates were not measured.

### Data analyses

Data were checked for normality and homogeneity of variance prior to analyses, and square-root or log-transformed, if necessary. Soil N pools, N-cycling processes and turnover times of N pools were analysed by mixed-effect model ANOVA with season and plant species as fixed factors, and block and sampling location nested within block (to account for the repeated-measures experimental design) as random factors. Additionally, we also ran models with season and plant functional type (PFT; here in the sense of shrubs versus moss) as fixed factors, and plant species, block and sampling location nested within block as random effects. We applied Tukey’s post-hoc tests for assessing differences between plant species over both seasons. In case of significant season x species interactions, post-hoc tests were run conditioned on season and plant species, respectively (Lenth 2024), i.e. species contrasts were estimated within single seasons and seasonal contrasts were estimated within species. Explained variance of mixed-effect models was calculated using marginal R^2^ (fixed effects only) and conditional R^2^ (fixed and random effects) (Nakagawa and Schielzeth 2013). Relationships between soil variables were estimated from Pearson correlation coefficients. All statistical analyses were performed using R version 3.5.1 (R Core Team 2018), with the packages ‘lmerTest’ (Kuznetsova et al. 2017), ‘emmeans’ (Lenth 2020), ‘MuMIn’ (Bartón 2016) and ‘Hmisc’ (Harrel 2019).

## Results

### Concentrations of plant available N-forms in soil

**Fig. 1:**
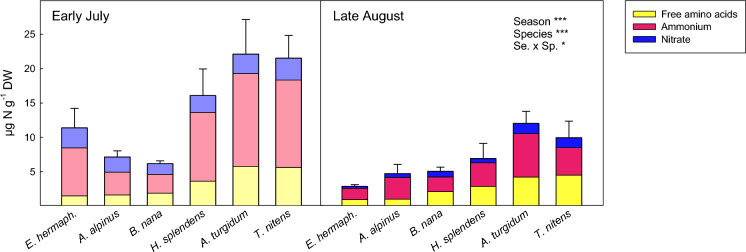
Concentrations of plant available N-forms in soil grown by the dwarf shrub species *Empetrum hermaphroditum, Arctostaphylos alpinus and Betula nana* and the moss species *Hylocomium splendens, Aulacomnium turgidum and Tomentypnum nitens* in early growing season (left panel) and late growing season (right panel). Error bars indicate 1 SE of total plant available N concentrations. *n* = 5. Effects of season, plant species and their interaction on total plant available N concentrations as determined by linear mixed effect models are indicated by ***(*p* < 0.001) and *(*p* < 0.05). For separate depiction of amino acid, ammonium and nitrate concentrations and standard errors see Fig. S1. Details on ANOVA models are presented in Table 2 and Table S1

The pool of extractable low molecular weight N compounds in soil was generally dominated by ammonium, followed by free amino acids and nitrate (Fig. 1). Moss soils were characterised by significantly higher concentrations of total free amino acids than shrub soils (PFT effect F_1,4_ = 22.94, p < 0.01, Table S1), with highest concentrations being found under the mosses *A. turgidum* and *T. nitens*, and lowest concentrations under the evergreen shrub *E. hermaphroditum* (Figs. 1, S1a). The availability of amino acids was slightly lower in late growing season than in early season (F_1,24_ = 13.74, p < 0.01, Table 2). Compared to the relatively minor seasonal contrasts in amino acids, we observed a pronounced seasonal effect on inorganic N availability (Fig. 1; Table 2), which, however, depended on plant functional types. While average NH_4_^+^ availability dropped by 60% from early to late growing season in moss soils (post-hoc test p < 0.001) and declined by 75% under the evergreen shrub *E. hermaphroditum* (post-hoc test p < 0.001), we found no seasonal differences in NH_4_^+^ availability at the deciduous shrub sites (Fig. S1b). Availability of NO_3_^−^ was significantly lower in late season than in early season at all sites (F_1,24_ = 50.97, p < 0.001, Table 2), but the difference was most pronounced at *E. hermaphroditum* sites, where NO_3_^−^ availability in late season on average accounted for only 10% of early season values (Fig. S1c).

**Table 2:** Summary of mixed-effect model ANOVA describing effects of seasonality and plant species on plant available soil N-pools, microbial N-cycling processes and turnover time of plant available N-pools

	Season (df = 1)	Plant species (df = 5)	Season x species (df = 5)	R^2^_m_	R^2^_c_
*Plant available N-pools*					
Free amino acids^b^	13.74**	15.04***	1.45	0.66	0.87
Ammonium^b^	41.21***	4.65**	3.12*	0.53	0.73
Nitrate^a^	50.97***	1.87	1.59	0.50	0.59
*Microbial N-cycling processes*					
Protein depolymerisation^b^	1.57	12.49***	1.05	0.55	0.83
Gross N mineralisation^a^	48.42***	2.09	2.90*	0.53	0.70
Net N mineralisation	18.92***	2.70*	0.97	0.32	0.57
Gross NH_4_^+^ consumption^a^	40.96***	2.33 ^+^	1.84	0.51	0.60
Gross nitrification^a^	0.01	3.62*	1.36	0.34	0.70
Net nitrification	25.18***	1.08	0.30	0.28	0.61
Gross NO_3_^−^ consumption^a^	8.91**	3.95**	0.81	0.38	0.71
*Turnover time of N-pools*					
Amino acids turnover^a^	5.53*	7.18***	0.47	0.35	0.64
Ammonium turnover^b^	3.32^+^	2.41^+^	2.11	0.34	0.37
Nitrate turnover^b^	24.64***	0.72	2.52^+^	0.26	0.74

Given are F-values for main effects and interaction. Significance levels: ***(p < 0.001), **(p < 0.01), *(p < 0.05) and + (p < 0.1). Explained variance by fixed effects (R^2^_m_) and including random effects (R^2^_c_)

^a^Square-root transformed data

^b^Log-transformed data

### Microbial N-cycling processes

**Fig. 2:**
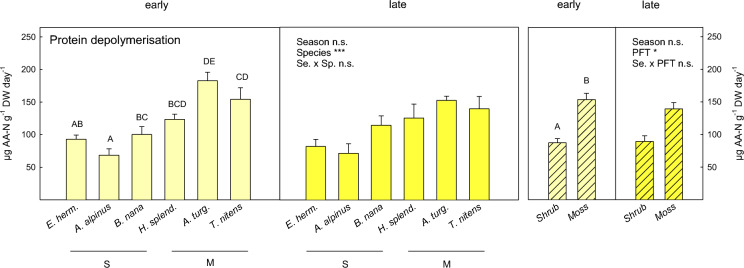
Protein depolymerisation rates in soil grown by the dwarf shrub species (S) *Empetrum hermaphroditum, Arctostaphylos alpinus and Betula nana* and the moss species (M) *Hylocomium splendens, Aulacomnium turgidum and Tomentypnum nitens* in early growing season (light yellow) and late growing season (dark yellow). Error bars indicate 1 SE. *n* = 5. Effects of season, plant species and their interaction determined by linear mixed effect models are indicated by ***(p < 0.001) and ‘n.s.’ (not significant). Uppercase letters indicate significant differences between plant species over both seasons by Tukey’s post-hoc test. Groups not sharing the same letter are significantly different (p < 0.05). Hatched bars present aggregated data of the three shrub and moss species, respectively. Significant effects of season and plant functional type (with plant species included as random factor in the model) are indicated by *(*p* < 0.05) and ‘n.s.’ (not significant). Details on ANOVA models are presented in Tables 2 and S1

Protein depolymerisation rates significantly differed among plant species and PFTs (Fig. 2; Table 2) and showed strong correlation with amino acid availability (r = 0.72, p < 0.001). Amino acid production rates in soils under the mosses *A. turgidum* and *T. nitens* were twice as high as under the ericaceous shrubs *E. hermaphroditum* and *A. alpinus* (post-hoc test p < 0.05). Differences in protein depolymerisation rates among plant species were, however, only marginally significant (F_5,24_ = 2.41, p = 0.066), if values were calculated per area, considering differences in bulk density and organic horizon depth, and did not show clear moss-shrub contrasts anymore (Table S2). There was no seasonal effect on proteolytic enzyme acidity (Fig. 2; Table 2).

**Fig. 3:**
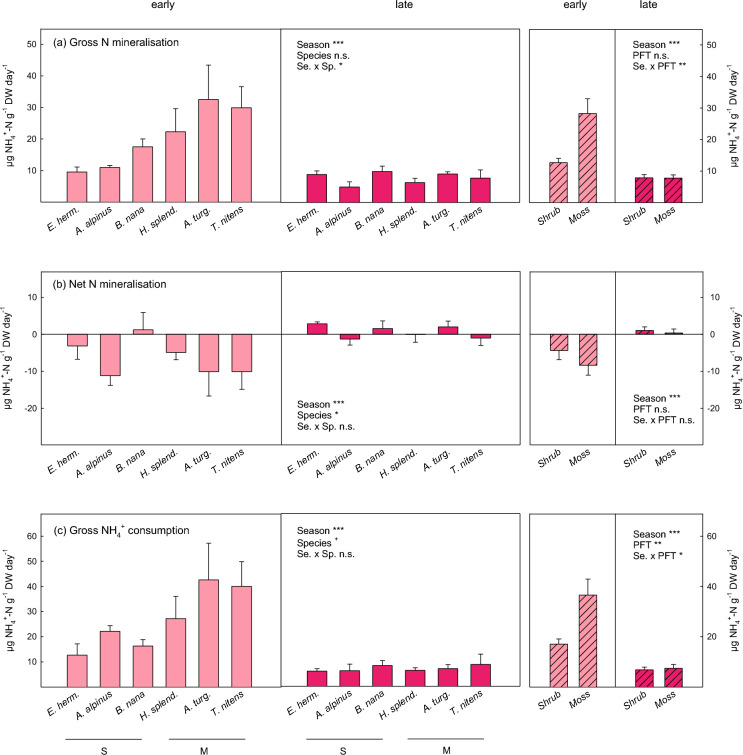
Gross N mineralisation rates (**a**), net N mineralisation rates (**b**) and gross ammonium consumption rates (**c**) in soil grown by the dwarf shrub species (S) *Empetrum hermaphroditum, Arctostaphylos alpinus and Betula nana* and the moss species (M) *Hylocomium splendens, Aulacomnium turgidum and Tomentypnum nitens* in early growing season (light pink) and late growing season (dark pink). Error bars indicate 1 SE. *n* = 4 (*E.h., A.a., B.n.*), *n* = 5 (*H.s., A.t., T.n.*). Effects of season, plant species and their interaction determined by linear mixed effect models are indicated by ***(*p* < 0.001), *(*p* < 0.05), ^+^(*p* < 0.1) and ‘n.s.’ (not significant). There were no significant post-hoc contrasts between plant species with *p* < 0.05 in Fig. 3b, despite a significant species effect in the ANOVA model. Hatched bars present aggregated data of the three shrub and moss species, respectively. Significant effects of season and plant functional type (with plant species included as random factor in the model) are indicated by ***(*p* < 0.001), **(*p* < 0.001), ^*^(*p* < 0.05) and ‘n.s.’ (not significant). Details on ANOVA models are presented in Tables 2 and S1

**Fig. 4:**
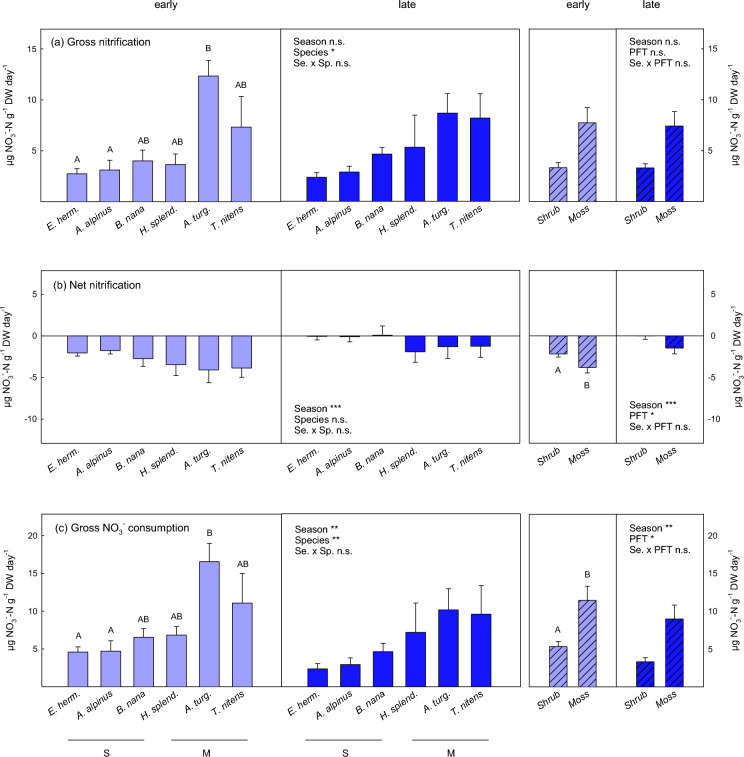
Gross nitrification rates (**a**), net nitrification rates (**b**) and gross nitrate consumption rates (**c**) in soil grown by the dwarf shrub species (S) *Empetrum hermaphroditum, Arctostaphylos alpinus and Betula nana* and the moss species (M) *Hylocomium splendens, Aulacomnium turgidum and Tomentypnum nitens* in early growing season (light blue) and late growing season (dark blue). Error bars indicate 1 SE. *n* = 5. Effects of season, plant species and their interaction determined by linear mixed effect models are indicated by ***(p < 0.001), **(p < 0.01), *(p < 0.05) and ‘n.s.’ (not significant). Uppercase letters indicate significant differences between plant species over both seasons by Tukey’s post-hoc test. Groups not sharing the same letter are significantly different (*p* < 0.05). Hatched bars present aggregated data of the three shrub and moss species, respectively. Significant effects of season and plant functional type (with plant species included as random factor in the model) are indicated by ***(*p* < 0.001), **(*p* < 0.001), *(*p* < 0.05) and ‘n.s.’ (not significant). Details on ANOVA models are presented in Tables 2 and S1

Gross N mineralisation rates, on the contrary, exhibited strong seasonal contrasts and significant plant species x season interactions (Fig. 3a; Table 2). The most dramatic seasonal change was observed in moss soils, where gross N mineralisation rates declined by more than 70% from early to late season (post hoc test p < 0.001). A significant decline in N mineralisation in late summer compared to early summer was also found at the deciduous shrub sites (*B. nana* and *A. alpinus)*, whereas no seasonal change was observed for the evergreen *E. hermaphroditum*. Net N mineralisation rates tended to be negative at the beginning of growing season, but were close to zero at the end of growing season (Fig. 3b), which resulted in even greater seasonal differences in gross NH_4_^+^ consumption rates than in gross N mineralisation rates (Fig. 3c). In contrast to gross N mineralisation, gross nitrification rates did not vary significantly between early and late season, but depended on the plant species (Fig. 4a; Table 2). Gross nitrification was greatest under the moss *A. turgidum*, and lowest under the evergreen shrub *E. hermaphroditum*. Gross nitrification rates were only weakly correlated with soil NH_4_^+^ availability (r = 0.29, p = 0.03) and uncorrelated with NO_3_^−^ availability (r = 0.11, p = 0.43). Similar to net N mineralisation, net nitrification was negative in early summer and close to zero in late summer (Fig. 4b). Moss soils generally exhibited more negative net nitrification than shrub soils. Gross NO_3_^−^ consumption significantly differed among plant species and was lower in late growing season than in early season (Fig. 4c).

### Turnover times of plant available N-pools

Estimated turnover times of plant available N-pools, calculated from the pool sizes and the production/consumption rates, revealed that the amino acid pool cycled most rapidly (Table 3). Turnover times of the amino acid pool ranged between 30 min and one hour, with generally slower turnover at moss sites than at shrub sites. Turnover of inorganic N compounds was considerably slower than that of amino acids, with turnover times between 3 h and more than one day, but opposite seasonal trends observed for ammonium and nitrate, respectively (Table 3): While estimated turnover of the ammonium pool tended to be slower in late summer than in early summer (with the exception of *E. hermaphroditum*), turnover of the nitrate pool was significantly faster at the end of growing season than at the beginning, except for *A. turgidum* sites.

**Table 3:** Estimated turnover time of plant available soil N-pools at sites grown by the dwarf shrub species *Empetrum hermaphroditum, Arctostaphylos alpinus and Betula nana* and the moss species *Hylocomium splendens, Aulacomnium turgidum and Tomentypnum nitens* in early and late growing season

Turnover time (h)	Free amino acids				Ammonium				Nitrate			
	Early season		Late season		Early season		Late season		Early season		Late season	
*E. hermaphr.*	0.4	(0.1)	0.3	(0.0) ^A^	28	(19)	6	(1)	20	(5)	3	(1)
*A. alpinus*	0.6	(0.2)	0.4	(0.1) ^AB^	5	(1)	15	(9)	17	(4)	4	(2)
*B. nana*	0.5	(0.1)	0.5	(0.1) ^AB^	4	(1)	6	(1)	8	(2)	5	(1)
*H. splendens*	0.7	(0.1)	0.6	(0.1) ^BC^	8	(3)	13	(5)	17	(9)	4	(2)
*A. turgidum*	0.8	(0.1)	0.7	(0.1) ^BC^	9	(2)	19	(2)	3	(0)	4	(1)
*T. nitens*	0.9	(0.1)	0.8	(0.1) ^C^	10	(2)	14	(3)	36	(21)	6	(2)
Seasonal effect	*p* < 0.05				*p* < 0.1				*p* < 0.001			

Values are means (SE in parentheses), n = 5, except for ammonium turnover time (*E.e, A.a., B.n., H.s*.) where n = 4. Uppercase letters indicate significant differences between plant species over both seasons by Tukey’s post-hoc test, groups not sharing the same letter are significantly different (p < 0.05)

## Discussion

This study aimed at enhancing our understanding of soil N cycling in tundra ecosystems by elucidating the effects of two dominant plant functional types in tundra heath, dwarf shrubs and mosses, on soil microbial N transformations and soil N availability. We investigated (1) how microbial gross N cycling processes and concentrations of plant available N forms in organic tundra soil vary in early and late growing season and (2) how these patterns are influenced by plant species / plant functional types. Our results demonstrate that the relative effects of seasonality, plant species and their interaction on soil N-cycling strongly depend on the specific N cycling process and the plant available N form considered.

### Protein depolymerisation

**Fig. 5:**
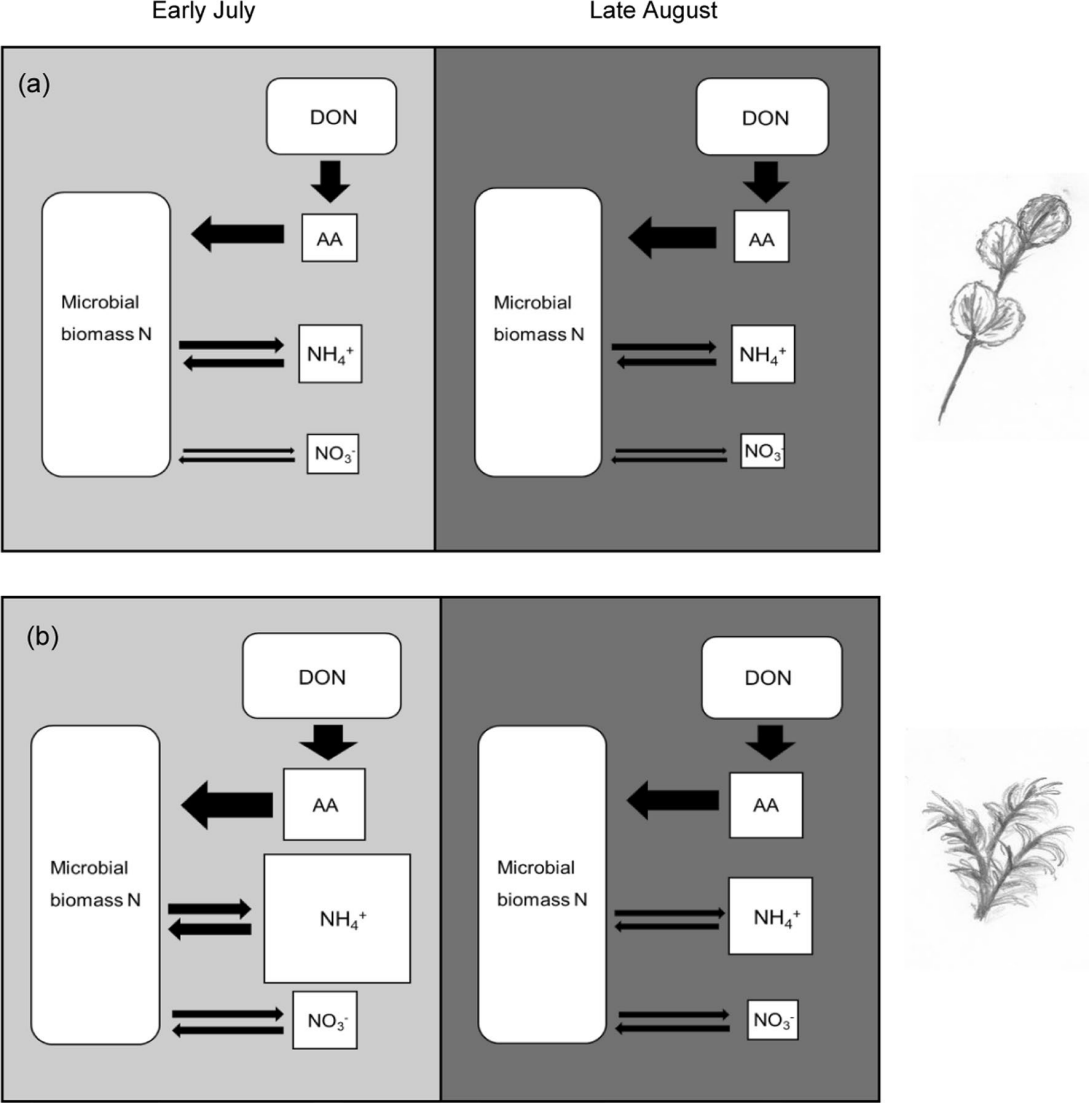
Schematic overview of microbial N transformations in soil under dwarf shrubs (*B. nana*; **a**) and mosses (*A. turgidum*; **b**) in early growing season (light grey panels) and late growing season (dark grey panels). The size of squares and thickness of arrows represents the pool sizes of plant available N-forms (amino acids, ammonium and nitrate, respectively) and the flux rates between the soil N pools (amino acid production and consumption, N mineralisation and ammonium immobilisation, nitrification and nitrate immobilisation). Amino acid consumption rates were estimated to be similar to production rates. Note that the sizes of the DON and microbial N pools are not proportional. (Microbial biomass N accounts for 50–100 times the pool size of total plant available N). Not depicted are ecosystems N losses (gaseous N losses and N leaching), which were not measured in this study. (Illustrations by M. Koranda)

Among the three microbial N cycling processes analysed in this study, gross protein depolymerisation was by far the greatest N flux, being one order of magnitude higher than gross N mineralisation and nitrification (Figs. 2, 5). The measured amino acid production rates in our study, which were determined by the toluene-addition method (see methods), are comparable to gross amino acid production rates in organic soils of Siberian tundra analysed by the ^15^N-pool dilution method (Wild et al. 2018). High amino acid production rates, but simultaneous rapid immobilisation of amino acids, predominantly by microbes and also plants (Schimel and Chapin 1996; Nordin et al. 2004; Clemmensen et al. 2008), lead to low levels of amino acid availability and high turnover rates of the amino acid pool (Fig. 1; Table 3), as previously reported from taiga forest soils (Jones and Kielland 2002; Kielland et al. 2007).

The strong contrasts in protein depolymerisation rates between shrub and moss soils at our study site (Fig. 2) were likely linked to plant species-related differences in the soil microbial community composition, soil organic matter (SOM) quality and soil pH-value, which were described in a previous study (Koranda et al. 2023). Specifically, moss soils were characterised by a bacterial-dominated microbial community, low soil C:N ratio and high soil pH-value, whereas shrub soils were characterised by a fungal-dominated microbial community, high soil C:N ratio and low soil pH (see also soil properties in Table 1). The high proteolytic activity at moss-grown sites was hence likely fuelled by fast turnover of microbial biomass N (the largest labile soil N pool) and soil protein, as well as lateral input of dissolved organic matter via subsurface water flow at the slightly wetter moss sites. Interestingly, and contrary to our expectations, we found that protein depolymerisation, the dominant soil N flux, did not vary significantly between early and late growing season, despite a 30% decrease in dissolved organic N (DON) availability in moss soils in late growing season. The temporal pattern of proteolytic activity rather followed potential protease activity, which was stable over the summer at the study site, as reported previously (Koranda et al. 2023). This suggests that protein depolymerisation in these organic soils was not substrate-limited, but rather enzyme-limited, i.e. regulated by the amount of protease in soil, as previously reported from Alaskan tundra soils (Weintraub and Schimel 2005b).

### N mineralisation

In contrast to protein depolymerisation, we observed strong seasonal changes in gross N mineralisation rates, which were dependent on the plant functional type (Fig. 3a). A short remark on what these rates actually measure may be useful beforehand, as there are frequent unclarities on this point. Gross N mineralisation rates are neither a measure of plant N availability (which lies between gross and net N mineralisation rate according to Booth et al. (2005)), nor do they directly reflect microbial N mining from SOM decomposition. Instead, gross N mineralisation rates are rather considered as a measure of internal N-cycling within the microbial community, reflecting the turnover of relatively labile microbial cellular N pools and microbial biomass (Fierer et al. 2001). Gross N mineralisation may be stimulated by both C and N addition, which may seem contradictory: While gross N mineralisation rates have been reported to generally correlate well with soil C availability (Booth et al. 2005; Buckeridge et al. 2010; Ramm et al. 2022a), increased N availability may enhance gross N mineralisation in N-poor soils by alleviating microbial N-limitation (Clein and Schimel 1995). The seasonal pattern in gross N mineralisation rates at moss and shrub sites in our study might in fact be explained by both of these mechanisms. In moss soils, high availability of DON, lasting from the spring peak in N availability, may have stimulated growth and turnover of the bacterial-dominated microbial community and hence gross N mineralisation in early growing season, in line with our first hypothesis, while a decline in DON availability at moss-grown sites over the summer lead to decreased microbial growth and N mineralisation rates. This pattern could also be linked to resource-driven shifts in the composition of the microbial community over the summer, i.e., increasing dominance of slow-growing oligotrophic microbial taxa in late growing season (Schmidt et al. 2007). In deciduous shrub soils, on the other hand, higher N mineralisation in early summer compared to late summer was probably related to the phase of intensive plant belowground C allocation and hence stimulation of the rhizosphere microbial community in early growing season, whereas at the end of summer this C flux had likely ceased in deciduous shrubs. This interpretation would also explain the different seasonal patterns at deciduous and evergreen shrub sites. Evergreen shrubs are known to exhibit generally low photosynthetic activity, which, however, persists over the entire snow-free season (Arndal et al. 2009), leading to low, but stable plant C supply to soil microbes and hence stable gross N mineralisation rates.

Our data on gross N-cycling rates also suggest that the concentrations of extractable low molecular weight N compounds in soil (e.g., the peak of inorganic N in early growing season, Fig. 1) do not necessarily allow to conclude on actual soil N availability. Early season soils were characterised by high gross N mineralisation, but at the same time exhibited negative net N mineralisation in some soils (Fig. 3b), suggesting high microbial N demand and generally faster N-cycling in early summer than in late summer. Although net N mineralisation and nitrification rates measured in our assays are not directly comparable to net rates measured in the field (as microbial N immobilisation may be stimulated by the conditions of our assays (Booth et al. 2005)), the significant seasonal contrasts in net N mineralisation and nitrification are nevertheless noteworthy.

### Nitrification

Despite the marked decline in NH_4_^+^ concentrations in late summer, which we observed in moss soils and under *E. hermaphroditum* (Fig. 1), gross nitrification rates were stable, except for a slight decrease at *A. turgidum* sites in late growing season (Fig. 4a). There are two possible explanations for this apparent contradiction: First, related to the point raised above, the ammonium concentrations measured in the soil extracts might not reflect the in-situ NH_4_^+^ availability for nitrifiers, which are known to be poor competitors against fast-growing heterotrophic microbes (Rasche et al. 2011). Second, nitrification might be regulated by abiotic and biotic factors other than NH_4_^+^ availability. The observed marked differences among plant species and the high spatial variation in nitrification rates rather point to the latter explanation. Gross nitrification rates in arctic soils have been reported to be strongly dependent on the community composition of nitrifier populations (dominated by ammonia-oxidizing archaea), which may have considerably greater impact on nitrification rates than N availability (Alves et al. 2013; Siljanen et al. 2019). The high nitrification rates at *the A. turgidum* sites may hence reflect microbial community characteristics driven by plant traits and related abiotic factors, e.g., high soil temperature fluctuations caused by the high thermal conductivity of *A. turgidum* moss cushions (Soudzilovskaia et al. 2013).

As gross nitrification rates were stable over the growing season at our study site, this leads to the question, what caused the marked decline in soil nitrate availability in late growing season (Fig. 1)? Both nitrate losses via denitrification or leaching would be possible causes. While denitrification may be substantial in permafrost peatlands (Marushchak et al. 2011; Voigt et al. 2020), gross nitrate consumption rates of our study (which were lower in late summer than in early summer) do not point to significant nitrate losses via denitrification under the conditions of our assay (Fig. 4c). Enhanced leaching losses in the field are hence the more plausible explanation for the low soil nitrate availability in late growing season. As in autumn thawing depth of permafrost soils is generally greatest, and discontinuous permafrost soils at our site were probably completely thawed, leaching losses of N may be significant (Treat et al. 2016), leading to significantly faster turnover of the nitrate pool than in early summer (Table 3).

### Drivers of soil N availability

It has been stated by Weintraub and Schimel (2005c) that N pool sizes in tundra soil are driven more by sink than by source processes. Our data suggest that this may depend on the N form, as well as on the microsite and the dominant plant species. The observed higher availability of amino acids at moss sites compared to shrub sites was likely source-driven, i.e., linked to differences in amino acid production rates. Amino acid immobilisation (which was not directly measured in our assay) was, however, obviously rapid at all sites, as amino acid concentrations were generally low despite high production rates. Our data hence corroborate findings by Wild et al. (2018) demonstrating that low N availability in organic tundra soil is not due to slow degradation of polymeric N substrates, but to fast (microbial) immobilisation of degradation products.

The strong seasonal contrasts in nitrate pool sizes were obviously sink-driven (likely by leaching losses). The seasonal variation in ammonium availability was probably source-driven at moss sites, following the decrease in microbial substrate availability and hence gross N mineralisation rates over the summer, but sink-driven at shrub sites. The pattern in inorganic N availability at shrub sites likely reflects differences in the timing of nutrient uptake between plant life forms, which is in accordance with our third hypothesis. In early summer, during the phase of intensive N-uptake of deciduous shrubs, inorganic N concentrations at *B. nana* and *A. alpinus* sites were lower than at *E. hermaphroditum sites*. The opposite, however, was the case in late growing season, reflecting relatively high N-uptake of evergreen shrubs at the end of growing season (Larsen et al. 2012).

When discussing contrasting effects of plant functional types on soil N availability, it is also relevant that all bryophyte species in our study are known to host N-fixing cyanobacteria, with especially high N-fixation rates found for *T. nitens* (Stuart et al. 2021). Associations with N-fixing bacteria provide essential N-supply for mosses in environments of low atmospheric N-deposition and may contribute significantly to long-term ecosystem N inputs (Lindo et al. 2013; Rousk et al. 2013; Holland-Moritz et al. 2021). The fresh N input via biological N-fixation may thus also have contributed to the elevated N-cycling rates in moss soils in our study. A comparison of the magnitude of reported N-fixing rates of moss-cyanobacteria associations with our measured soil N-cycling rates, however, shows, that moss N-fixation is unlikely to be the main direct cause for the observed pattern in soil N availability. Average amino acid production rate in the organic horizon in our study was 0.95 g N m^−2^ day^−1^ in moss soils and 0.82 g N m^−2^ day^−1^ in shrub soils (Table S2), whereas reported moss N-fixation rates in high-latitude ecosystems range from less than 0.01 to 0.9 g N m^−2^ year^−1^ (Lindo et al. 2013; Rousk and Michelsen 2017). It should be noted that the N-cycling rates presented in our study are not in-situ rates and may hence be overestimated. However, the fact that the greatest soil N-flux, i.e. amino acid production via soil proteolytic activity, is orders of magnitude higher than the N input via N-fixation, suggests that the observed contrasts in soil N availability between moss and shrub sites are likely not a direct effect of biological N-fixation but rather reflect differences in activity of heterotrophic soil microbes, i.e., decomposition of SOM and fresh DOM, as well as turnover of microbial biomass.

Finally, it should be noted that the observational approach, which we used for investigating effects of plant functional types on soil N-cycling in this study, does not allow to clearly distinguish effects of plant species on soil microbial processes from effects related to site-preferences of plant species (e.g., differences in subsurface water flow). The strength of this approach, however, is that it elucidates not only short-term plant-soil-microbe interactions via plant nutrient uptake and belowground C allocation, but also allows to investigate, under undisturbed conditions, the long-term impact of plant species and plant functional types on soil N-cycling via effects on SOM quality and soil microbial community composition, which is crucial in high-latitude ecosystems characterised by very slow plant growth, litter decomposition and soil formation.

## Conclusions

Our study revealed three main findings (see overview in Fig. 5):

1. Moss soils were characterised by significantly higher N cycling rates and soil N availability than shrub soils, which was likely linked with a bacterial-dominated microbial community and low soil C:N ratio of moss soils.
2. Protein depolymerisation, the dominant soil N flux, as well as gross nitrification rates, did not vary significantly between early and late growing season, whereas gross N mineralisation and inorganic N availability markedly dropped in late summer at most sites.
3. The magnitude of seasonal changes in soil N cycling strongly differed among plant functional types: The decline in gross N mineralisation and NH_4_^+^ availability in late summer was most pronounced in moss soils, presumably caused by a decrease in substrate availability and microbial community changes over the growing season. Furthermore, deciduous and evergreen shrub soils, respectively, exhibited distinct seasonal patterns in gross N mineralisation rates and inorganic N concentrations, likely reflecting differences in timing of plant nutrient uptake and photosynthetic activity between plant growth forms.

Our results hence demonstrate that the spatial variation and seasonal dynamics of microbial N transformations and soil N availability in tundra heath are intimately linked with the distinct influence of plant functional types on soil microbial community composition and activity and with the plant species-specific patterns of nutrient uptake and photosynthetic activity. This suggests potential strong impacts of future global change-induced shifts in plant community composition on soil N cycling in tundra ecosystems. E.g., it can be expected that the expansion of deciduous shrubs like *B. nana* into moss dominated areas will reduce the intensity and duration of the spring peak in soil N availability and hence lower the potential for gaseous ecosystem N losses in early growing season. This may have major implications for ecosystem N budget and cause feedback on climate via influencing atmospheric concentrations of the potent greenhouse gas nitrous oxide. This example demonstrates that the close coupling of plant functional types with soil microbial communities ultimately drives the seasonal and spatial dynamics of soil N-cycling and N-availability in tundra ecosystems.

## Supplementary Information

Below is the link to the electronic supplementary material.

Supplementary file1 (PDF 240 kb)

## Data Availability

The data that support the findings of this study are available at the ‘Mendeley Data’ repository (https://data.mendeley.com/datasets/vy6crrtywn/1).
