# Supplementary material for: Microbial nitrogen transformations in tundra soil depend on interactive effects of seasonality and plant functional types
Source: Biogeochemistry. 2024 Aug 30;167(11):1391–408. doi: 10.1007/s10533-024-01176-6 (PMC11564215; doi:10.1007/s10533-024-01176-6)
Supplement: Supplementary file 1 — Supplementary file1 (PDF 240 kb) [file 10533_2024_1176_MOESM1_ESM.pdf]

Supporting Information to the article 'Microbial nitrogen transformations in tundra soil depend on interactive effects of seasonality and plant functional types'

Biogeochemistry (2024)

Marianne Koranda * and Anders Michelsen

* Corresponding author: M. Koranda, Division of Terrestrial Ecosystem Research, Centre for Microbiology and Environmental Systems Science, University of Vienna, 1030 Vienna, Austria. E-mail: marianne.koranda@univie.ac.at.

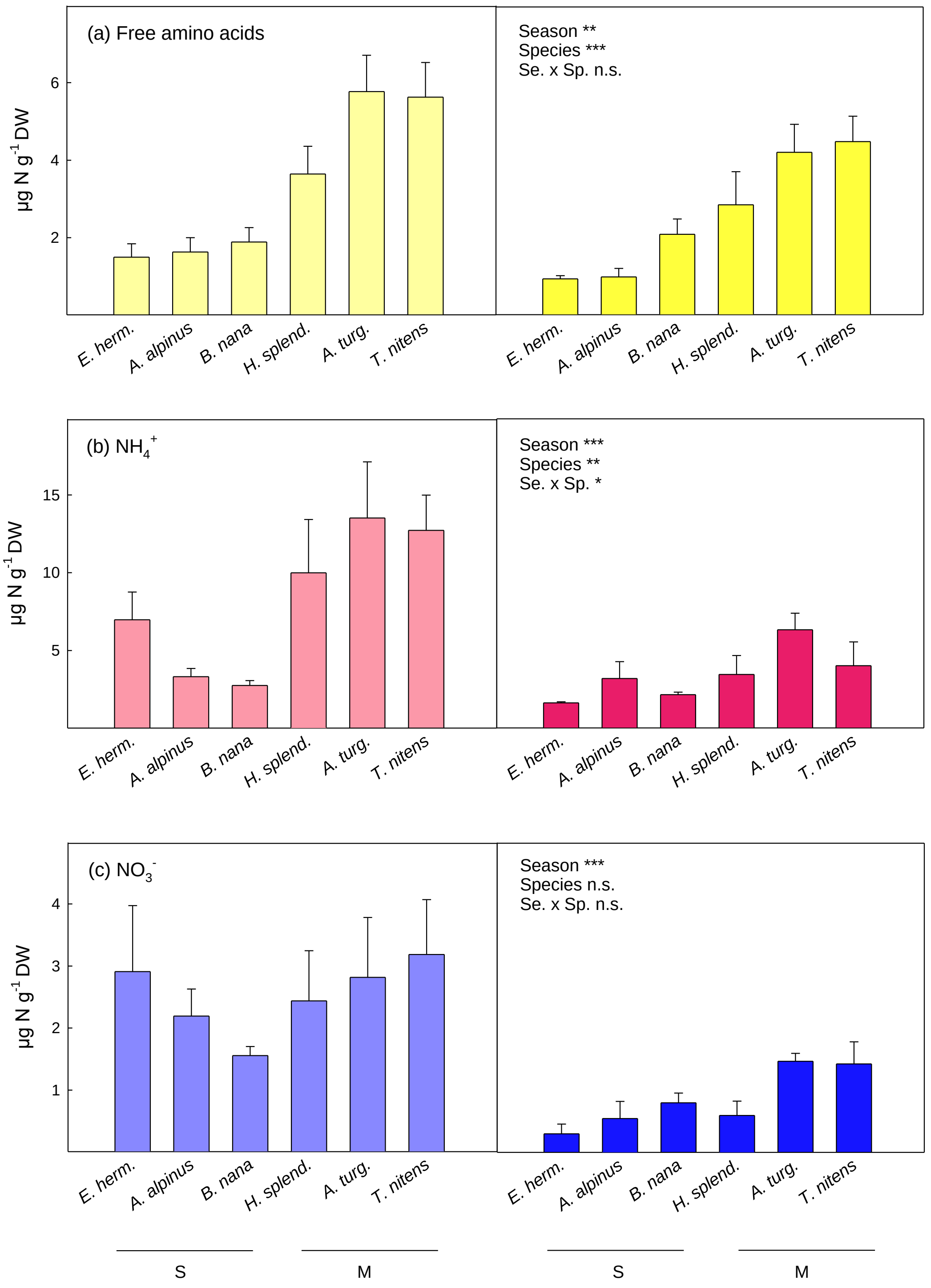

Fig. S1 Concentrations of free amino acids (a), ammonium (b) and nitrate (c) in soil grown by the dwarf shrub species (S) *Empetrum hermaphroditum, Arctostaphylos alpinus and Betula nana* and the moss species (M) *Hylocomium splendens, Aulacomnium turgidum and Tomentypnum nitens* in early growing season (left panels) and late growing season (right panels). Error bars indicate 1 SE. n = 5. Effects of season, plant species and their interaction determined by linear mixed effect models are indicated by *** ($p<0.001$), ** ($p<0.01$), * ($p<0.05$) and 'n.s.' (not significant). Details on ANOVA models are presented in Table 2 and Table S1.

Table S1 Summary of mixed-effect model ANOVA describing effects of seasonality and plant functional type (PFT, i.e. shrubs versus mosses) on plant available soil N-pools and microbial N-cycling processes.

	Season (df = 1)	PFT (df = 1)	PFT x season (df = 1)	R^2_m	R^2_c
**Plant available N-pools**					
Free amino acids [b]	12.31 **	22.94 **	0.03	0.56	0.85
Ammonium [b]	31.96 ***	17.54 *	2.14	0.44	0.61
Nitrate [a]	45.85 ***	4.78 +	0.75	0.44	0.51
**Microbial N-cycling processes**					
Protein depolymerisation [b]	1.57	10.49 *	1.24	0.39	0.83
Gross N mineralisation [a]	50.73 ***	3.81	11.91 **	0.48	0.69
Net N mineralisation	19.33 ***	2.06	1.35	0.23	0.56
Gross NH_4^+ consumption [a]	42.08 ***	8.59 **	5.92 *	0.49	0.59
Gross nitrification [a]	0.05	3.76	0.00	0.16	0.62
Net nitrification	28.31 ***	6.22 *	0.08	0.30	0.61
Gross NO_3^- consumption [a]	8.18 **	8.79 *	0.01	0.28	0.66

Given are F-values for main effects and interaction. Plant species is included as random effect in the models. Significance levels: *** (p<0.001), ** (p<0.01), * (p<0.05) and + (p<0.1). Explained variance by fixed effects (R^2_m) and including random effects (R^2_c). [a] Square-root transformed data. [b] Log-transformed data.

Table S2 (A) Plant available N-forms and (B) microbial N-transformation rates per area at sites grown by the dwarf shrub species *Empetrum hermaphroditum, Arctostaphylos alpinus and Betula nana* and the moss species *Hylocomium splendens, Aulacomnium turgidum and Tomentypnum nitens* in early growing season and late growing season.

(A)

(mg N m^{-2})	Free amino acids		Ammonium		Nitrate	
	Early season	Late season	Early season	Late season	Early season	Late season
E. hermaphr.	12.7 (3.4)	7.8 (0.8) [A]	57.1 (12.5)	13.4 (0.6)	22.8 (6.7)	2.4 (1.2)
A. alpinus	16.6 (4.2)	10.0 (2.7) [AB]	32.9 (5.6)	32.0 (11.7)	21.6 (4.5)	5.8 (3.0)
B. nana	19.6 (5.7)	22.2 (7.4) [ABC]	24.8 (1.5)	21.1 (4.5)	14.4 (1.4)	8.4 (2.4)
H. splendens	32.0 (7.9)	25.6 (8.5) [BC]	95.2 (37.5)	29.4 (10.9)	23.0 (10.4)	5.4 (2.2)
A. turgidum	33.5 (7.1)	24.2 (5.0) [BC]	78.8 (21.9)	36.9 (7.7)	16.2 (5.9)	8.6 (1.4)
T. nitens	32.1 (2.8)	26.4 (2.8) [C]	75.6 (11.1)	23.6 (8.3)	18.7 (5.0)	8.7 (2.1)
Seasonal effect	$p < 0.01$		$p < 0.001$		$p < 0.001$	

Values are means (SE in parentheses), n = 5. Uppercase letters indicate significant differences between plant species over both seasons after Tukey's post-hoc test. Groups not sharing the same letter are significantly different ($p < 0.05$). Depth of organic horizon and bulk density for each plant species is given in Table 1.

(B)

(g N m^{-2} day^{-1})	Protein depolymerisation	
	Early season	Late season
E. hermaphr.	0.77 (0.03)	0.67 (0.08)
A. alpinus	0.68 (0.11)	0.68 (0.09)
B. nana	0.98 (0.22)	1.09 (0.19)
H. splendens	1.04 (0.06)	1.05 (0.16)
A. turgidum	1.02 (0.09)	0.86 (0.09)
T. nitens	0.91 (0.07)	0.82 (0.06)

Seasonal effect	*n.s.*

($g\ N\ m^{-2}\ day^{-1}$)	Gross N mineralisation				Net N mineralisation				Gross NH_4^+ consumption			
	Early season		Late season		Early season		Late season		Early season		Late season	
E. hermaphr.	0.08	(0.01)	0.07	(0.01)	-0.03	(0.03)	0.02	(0.00) AB	0.11	(0.04)	0.05	(0.01)
A. alpinus	0.12	(0.01)	0.05	(0.02)	-0.12	(0.03)	-0.01	(0.02) A	0.23	(0.02)	0.07	(0.03)
B. nana	0.18	(0.04)	0.10	(0.01)	0.01	(0.04)	0.01	(0.02) B	0.18	(0.04)	0.09	(0.03)
H. splendens	0.19	(0.06)	0.06	(0.01)	-0.05	(0.02)	0.00	(0.02) AB	0.24	(0.08)	0.06	(0.01)
A. turgidum	0.19	(0.07)	0.05	(0.01)	-0.05	(0.04)	0.01	(0.01) AB	0.24	(0.09)	0.04	(0.01)
T. nitens	0.18	(0.03)	0.05	(0.01)	-0.06	(0.02)	0.00	(0.01) AB	0.24	(0.05)	0.05	(0.02)
Seasonal effect	$p < 0.001$				$p < 0.001$				$p < 0.001$			

($g\ N\ m^{-2}\ day^{-1}$)	Gross nitrification				Net nitrification				Gross NO_3^- consumption			
	Early season		Late season		Early season		Late season		Early season		Late season	
E. hermaphr.	0.021	(0.002)	0.019	(0.003)	-0.017	(0.004)	-0.001	(0.004)	0.037	(0.004)	0.020	(0.005)
A. alpinus	0.032	(0.011)	0.030	(0.007)	-0.018	(0.005)	0.000	(0.007)	0.048	(0.016)	0.030	(0.010)
B. nana	0.042	(0.012)	0.046	(0.010)	-0.024	(0.008)	-0.001	(0.012)	0.065	(0.014)	0.048	(0.016)
H. splendens	0.031	(0.009)	0.048	(0.028)	-0.027	(0.008)	-0.016	(0.009)	0.056	(0.009)	0.063	(0.035)
A. turgidum	0.070	(0.013)	0.051	(0.013)	-0.021	(0.007)	-0.008	(0.007)	0.092	(0.015)	0.060	(0.017)
T. nitens	0.047	(0.021)	0.046	(0.009)	-0.022	(0.004)	-0.005	(0.006)	0.069	(0.025)	0.051	(0.012)
Seasonal effect	*n.s.*				$p < 0.001$				$p < 0.01$			

Values are means (SE in parentheses), n = 5, except for N mineralisation / NH_4^+ consumption (*E.e, A.a., B.n., H.s.*) where n = 4. Uppercase letters indicate significant differences between plant species over both seasons after Tukey's post-hoc test, groups not sharing the same letter are significantly different ($p < 0.05$). Depth of organic horizon and bulk density for each plant species is given in Table 1.
